# Supplementary material for: Identification of photoperiod-induced specific miRNAs in the adrenal glands of Sunite sheep (Ovis aries)
Source: Front Vet Sci. 2022 Jul 22;9:888207. doi: 10.3389/fvets.2022.888207 (PMC9354845; doi:10.3389/fvets.2022.888207)
Supplement: Supplementary file 1 [file Data_Sheet_1.ZIP › Supplementary file/miRDeep2 file.zip!/miRDeep2 file/Know_Structure/SPLP42A1/oar-mir-329b.pdf]

miRBase precursor : oar-mir-329b
Total read count : 2392
oar-miR-329b-5p read count : 22
oar-miR-329b-3p read count : 2352
remaining reads : 18

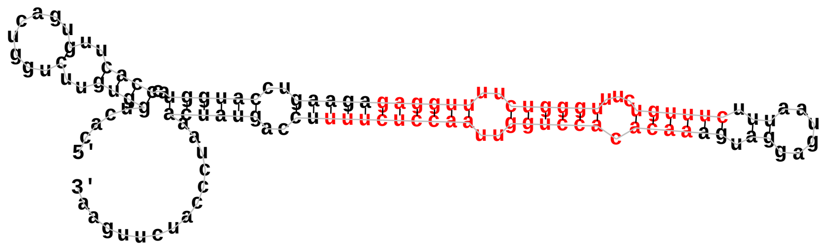

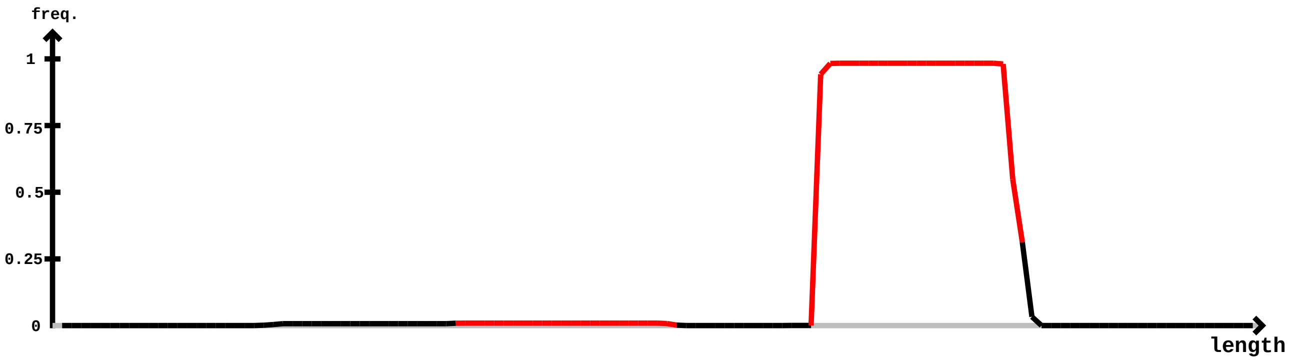

oar-miR-329b-3p

oar-miR-329b-5p

5'- cacugguguucuggucaguguucacccaugguaccugaagagagguuuucuggguuucuguuucuuuaaugaggaugaaacacaccugguuaaccucuuuuccaguaucaaaucccaucuugaa -3'	exp	
....((((..(........)..))))..((((((..((((((((((..((((((...((((((.((......)).)))))).))))))..))))))))))..))))))................	reads	mm
....................uucacccaugguaccugaaga...	3	0
.....................ucacccaugguaccugaaga...	6	0
......................cacccaugguaccugaaga...	8	0
....agagguuuucuggguuucuguu..	2	0
....agagguuuucuggguuucuguuu...	2	0
....gagguuuucuggguuucuguu..	2	0
....gagguuuucuggguuucuguuu...	12	0
....gagguuuucuggguuucuguuuc..	4	0
....ugaaacacaccugguuaaccucu..........................	1	0
....aacacaccugguuaaccuc...........................	7	0
....aacacaccugguuaaccucu..........................	1027	0
....aacacaccugguuaaccucuu.........................	558	0
....aacacaccugguuaaccucuuu........................	632	0
....aacacaccugguuaaccucuuuu.......................	29	0
....acacaccugguuaaccucu..........................	4	0
....acacaccugguuaaccucuu.........................	8	0
....acacaccugguuaaccucuuu........................	35	0
....acacaccugguuaaccucuuuu.......................	50	0
....cacaccugguuaaccucu..........................	1	0
....cacaccugguuaaccucuu.........................	1	0
